# Empirical superior vena cava isolation improves outcomes of radiofrequency re-ablation in pulmonary vein isolation non-responders: A 2-center retrospective study in China

**DOI:** 10.3389/fcvm.2022.1049414

**Published:** 2022-12-07

**Authors:** Zhoushan Gu, Gang Yang, Weizhu Ju, Mingfang Li, Hongwu Chen, Kai Gu, Hailei Liu, Minglong Chen

**Affiliations:** ^1^Department of Cardiology, Affiliated Hospital of Nantong University, Nantong, China; ^2^Department of Cardiology, The First Affiliated Hospital of Nanjing Medical University, Nanjing, China

**Keywords:** atrial fibrillation, recurrence, pulmonary vein isolated, non-PV trigger, superior vena cava

## Abstract

**Background:**

Pulmonary vein isolation (PVI) is the standard ablation strategy for treating atrial fibrillation (AF). However, the optimal strategy of a repeat procedure for PVI non-responders remains unclear.

**Objective:**

This study aims to investigate the incidence of PVI non-responders in patients undergoing a repeat procedure, as well as the predictors for the recurrence of repeat ablation.

**Methods:**

A total of 276 consecutive patients who underwent repeat ablation from August 2016 to July 2019 in two centers were screened. A total of 64 (22%) patients with durable PVI were enrolled. Techniques such as low voltage zone modification, linear ablation, non-PV trigger ablation, and empirical superior vena cava (SVC) isolation were conducted.

**Results:**

After the 20.0 ± 9.9 month follow-up, 42 (65.6%) patients were free from atrial arrhythmias. A significant difference was reported between the recurrent and non-recurrent groups in non-paroxysmal AF (50 vs. 23.8%, *p* = 0.038), diabetes mellitus (27.3 vs. 4.8%, *p* = 0.02), and empirical superior vena cava (SVC) isolation (28.6 vs. 60.5%, *p* = 0.019). Multivariate regression analysis demonstrated that empirical SVC isolation was an independent predictor of freedom from recurrence (95% CI: 1.64–32.8, *p* = 0.009). Kaplan-Meier curve demonstrates significant difference in recurrence between empirical and non-empirical SVC isolation groups (HR: 0.338; 95% CI: 0.131–0.873; *p* = 0.025).

**Conclusion:**

About 22% of patients in repeat procedures were PVI non-responders. Non-paroxysmal AF and diabetes mellitus were associated with recurrence post-re-ablation. Empirical SVC isolation could potentially improve the outcome of repeat procedures in PVI non-responders.

## Introduction

Atrial fibrillation (AF) is among the most common arrhythmias in clinical practice. Pulmonary vein isolation (PVI) has become the cornerstone of ablation for paroxysmal and non-paroxysmal AF since Haissaguerre demonstrated that AF primarily originated from pulmonary veins (PVs) (1, 2). Despite the continuous development of ablation strategies and techniques, many patients continue to suffer from atrial arrhythmias recurrence post initial radiofrequency catheter ablation (RFCA) (3). PV reconnection was considered to be the primary cause for the recurrence of AF (4). However, the PVs in some patients were still isolated during the second procedure (5, 6). Empirical strategies of the second ablation in these patients comprise left atrial substrate modification, ablation of non-PV triggers, and linear ablation (7, 8). The optimal strategy of the repeat procedure for PVI non-responders remains unclear. This study investigates the incidence of PVI non-responders in patients undergoing repeat procedures and the predictors for the recurrence of repeat ablation.

## Materials and methods

### Study population

From August 2016 to July 2019, patients with recurrent AF who underwent first repeat ablation in The First Affiliated Hospital of Nanjing Medical University and Affiliated Hospital of Nantong University were screened. The inclusion criteria considered durable PVI in a repeat procedure. The exclusion criteria comprised: (1) patients underwent multiple ablations for AF, (2) the index ablation with cryoballoon, and (3) surgical ablation. An informed consent form of RFCA was signed before the procedure. The study was approved by the ethics committee of our institute.

### Ablation strategies and techniques

#### Fibrotic modification based on voltage mapping during sinus rhythm

Ablation of fibrosis identified by voltage mapping was the primary strategy for PVI non-responders. If the initial rhythm was AF, cardioversion was initially taken. A high-density voltage mapping of the left atrium (LA) during SR was performed, using three-dimensional mapping systems (Carto, Ensite, or Rhythmia) and high-density mapping catheters (Pentaray, AFocus II, or Orion). A low voltage zone was identified as the bipolar voltage in the 0.1–0.4 mV range. Fibrosis-based modification was then performed to achieve an absolute bipolar electrogram of <0.1 mV. The detailed steps are prescribed in our prior study (9).

#### Linear ablation and its bidirectional block

If macro-reentry atrial tachycardia occurred spontaneously or was induced during the procedure, linear ablation was performed to terminate it. Another linear ablation was added based on the discretion of the operating physician, including the anterior wall line, roof line, posterior wall line, and cavotricuspid isthmus line. A bidirectional block was validated as the endpoint of linear ablation.

#### Non-PV triggers elimination

Non-PV triggers were provoked in all patients. After left atrium ablation, an intravenous infusion of isoproterenol was administered to increase the heart rate by 30%, followed by a bolus injection of adenosine triphosphate (20–40 mg) to provoke non-PV triggers. All revealed non-PV triggers were targeted.

#### Empirical superior vena cava isolation

SVC was the most common non-PV trigger source (10). If SVC was confirmed as a trigger source by drug provocation, SVC isolation was performed. Otherwise, empirical SVC isolation was at the discretion of the physician. RA-SVC angiography was performed by manual injection and the junction of the convex RA wall and the straight SVC wall was defined as the radiological RA-SVC junction (2). Electroanatomic maps of RA and the region around the radiologic RA-SVC junction were created during sinus rhythm, and the earliest activation region was defined as the sinus node area (3). The right phrenic nerve was mapped subsequently by pacing around the RA-SVC with an output of 20 mA (4). Point-by-point ablation was delivered at the level of 1–2 cm above the RA-SVC junction with 20–25 s for each point, and RF energy was set as 20–30 W with 17 ml/min of saline irrigation and a maximum temperature of 42°C using an open irrigation catheter. Great carefulness was taken to avoid injury to sinus node and phrenic nerve (5). The ablation endpoint was set as bidirectional block across the line.

### Follow-up

Anti-arrhythmic drugs were discontinued 3 months after the procedure. Patients were followed up at 1, 3, 6, and 12 months and then yearly post the repeat procedure. 24-h or 7-day Holter ECGs were recorded at every visit. An ECG was performed in case of any symptoms of palpitation. Recurrence was defined as any atrial tachyarrhythmia that lasted longer than 30 s after the 3-month blank period.

### Statistical analysis

Continuous variables were described as mean ± standard deviation values, and a *t*-test was performed to compare the two groups. Categorical variables were expressed as numbers and percentages. The chi-square or Fisher’s exact test were used to compare the categorical data. To identify the factors associated with recurrence, multivariate logistic regression was performed (Forward LR Method) with the variables of statistical significance in univariate analysis. Statistical analysis was performed using SPSS 26. A *p*-value < 0.05 was considered statistically significant.

## Results

**FIGURE 1:**
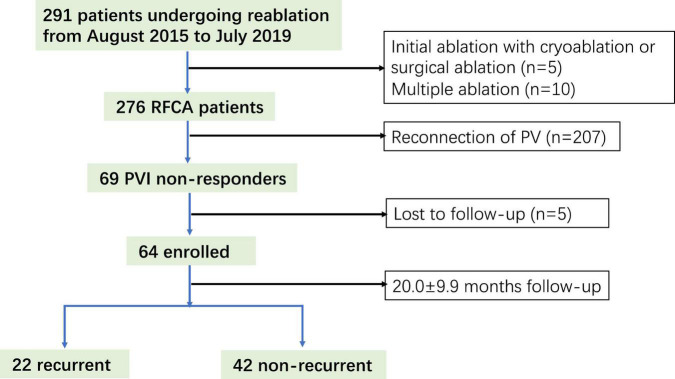
Study flow diagram depicting the patient enrollment. RFCA, radiofrequency catheter ablation.

A total of 291 patients undergoing re-ablation for AF were screened, of which 64 (22%) (age; 60.7 ± 11.0 years; 35 men) were PVI non-responders and enrolled in this study (Figure 1). After the 20.0 ± 9.9 month follow-up, 42 (65.6%) patients were free from atrial arrhythmias. A significant difference was observed between the recurrent and non-recurrent groups in non-paroxysmal AF (50 vs. 23.8%, *p* = 0.038) and diabetes mellitus (27.3 vs. 4.8%, *p* = 0.02). The demographic and clinical characteristics are illustrated in Table 1.

**TABLE 1:** Demographic and clinical characteristics of the recurrent and non-recurrent groups.

	Recurrent group (*n* = 22)	Non-recurrent group (*n* = 42)	*p*-value
Age (years)	62.2 ± 12.4	59.9 ± 10.3	0.421
Male *n* (%)	11 (50)	24 (57.1)	0.586
BMI (kg/m^2^)	25.4 ± 2.2	26.1 ± 3.0	0.36
Persistent AF *n* (%)	11 (50)	10 (23.8)	0.038
AF history duration (years)	5.8 ± 4.6	6.8 ± 4.4	0.386
Time to recurrence (years)	1.6 ± 0.9	2.4 ± 1.9	0.073
**Co-morbidity**			
Hypertension *n* (%)break	12 (54.5)	17 (40.5)	0.285
Diabetes *n* (%)	6 (27.3)	2 (4.8)	0.02
CHD *n* (%)	4 (18.2)	2 (4.8)	0.102
CHA2DS2-VASc	2.1 ± 1.7	1.3 ± 1.1	0.051
HASBLED	1.5 ± 0.9	0.9 ± 0.8	0.105
**Echocardiogram**			
LAD (mm)	40.3 ± 4.8	38.7 ± 5.9	0.305
LVDd (mm)	49.0 ± 6.2	46.4 ± 3.6	0.065
LVEF (%)	62.1 ± 5.4	63.3 ± 3.4	0.344

AF, atrial fibrillation; BMI, body mass index; CHD, coronary heart disease; LAD, left atrial dimeter; LVDd, left ventricular diastolic dimeter; LVEF, left ventricular ejection fraction; PV, pulmonary vein; SVC, superior vena cava.

### Procedural characteristics and outcomes

#### Prevalence and distribution of low voltage areas

**FIGURE 2:**
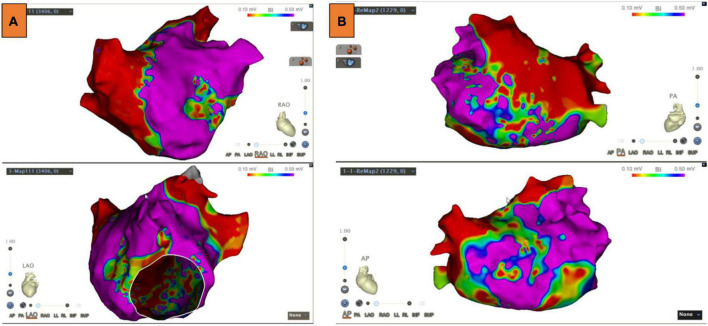
Low voltage areas (LVAs) of the left atrium (LA). **(A)** LVAs were detected in the anterior wall of LA. **(B)** LVAs were detected in the roof and septal wall of LA. Substrate modification was performed in these areas, and an absolute bipolar electrogram of <0.1 mV was achieved.

LA voltage maps were created in all patients with 1,324 ± 335 mapping points per patient. Significant LVAs within the LA were found in 33 (51.6%) patients. Among these patients, the septum was involved in 6.3% of cases, the anterior LA in 45.3%, the posterior wall in 14.1%, and the atrial roof in 20.3% (Figure 2). Substrate modification was performed in LVAs.

#### Linear ablation

Spontaneous or induced ATs occurred in 28 (43.8%) patients. Among these ATs, 7 (25%) were roof-dependent macro-reentry, 12 (42.9%) were peri-mitral atrial flutters, 4 (14.3%) were counterclockwise atrial flutters, and 5 (17.9%) were focal. Linear ablation was performed in 31 patients, including 23 anterior walls linear ablation, 8 posterior walls linear ablation, 14 LA roof linear ablation, and 22 CTI linear ablation. A bidirectional block was achieved as the endpoint of linear ablation.

#### Non-PV triggers

Non-PV triggers were confirmed in nine patients (14.1%). Among them, five cases (7.8%) in SVC, three (4.7%) in fossae ovalis, and two (3.2%) in crista terminalis. In 59 patients without definite SVC triggers, empirical SVC isolation was performed in 29 patients. The demographic and clinical characteristics of empirical and non-empirical SVC isolation groups are illustrated in Table 2.

**TABLE 2:** Demographic and clinical characteristics of empirical and non-empirical superior vena cava (SVC) isolation groups.

	Empirical SVC isolation (*n* = 29)	Non-empirical SVC isolation (*n* = 30)	*p*-value
Age (years)	61.5 ± 9.2	60.1 ± 13.2	0.642
Male *n* (%)	19 (65.5)	15 (50.0)	0.228
BMI (kg/m^2^)	24.8 ± 2.1	26.9 ± 3.0	0.003
Persistent AF *n* (%)	9 (31.0)	12 (40.0)	0.472
AF history duration (years)	6.9 ± 4.0	6.0 ± 4.4	0.435
Time to recurrence (years)	2.5 ± 1.9	1.9 ± 1.6	0.213
**Co-morbidity**			
Hypertension *n* (%)	11 (37.9)	17 (56.7)	0.150
Diabetes *n* (%)	1 (3.4)	7 (23.3)	0.026
CHD *n* (%)	2 (6.9)	4 (13.3)	0.413
CHA2DS2-VASc	1.2 ± 1.1	1.9 ± 1.6	0.043
HASBLED	0.9 ± 0.8	1.2 ± 1.0	0.113
**Echocardiogram**			
LAD (mm)	38.6 ± 4.9	41.0 ± 5.8	0.106
LVDd (mm)	46.9 ± 3.2	48.3 ± 6.1	0.297
LVEF (%)	63.6 ± 3.0	62.0 ± 5.4	0.170

AF, atrial fibrillation; BMI, body mass index; CHD, coronary heart disease; LAD, left atrial dimeter; LVDd, left ventricular diastolic dimeter; LVEF, left ventricular ejection fraction; PV, pulmonary vein; SVC, superior vena cava.

#### Outcomes of repeat procedure

**FIGURE 3:**
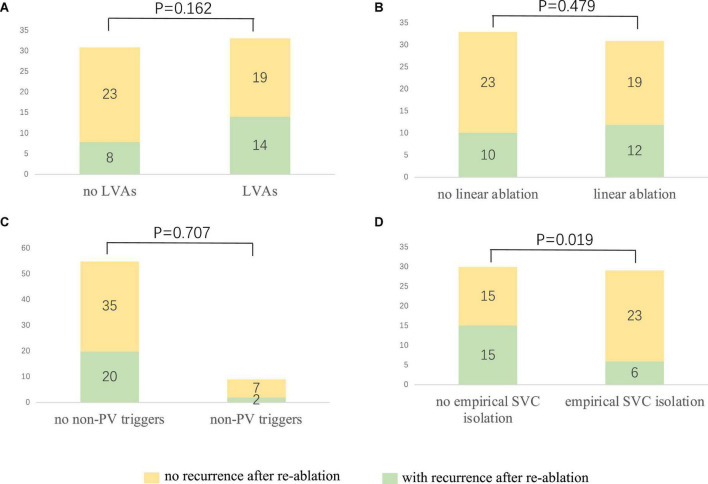
The relationship between procedure characteristics and the re-ablation outcome. **(A)** No significant difference could be observed among the patients with and without LVAs (42.4 vs. 25.8%, *p* = 0.162). **(B)** Linear ablation did not improve the outcome of the redo procedure (38.7 vs. 30.3%, *p* = 0.479). **(C)** No significant difference could be identified among the patients with and without confirmed non-PV triggers (36.4 vs. 22.2%, *p* = 0.707). **(D)** Empirical SVC isolation improved the re-ablation outcome (20.7 vs. 50%, *p* = 0.019). LVA, low voltage area; PV: pulmonary vein; SVC, superior vena cava.

After a 20.0 ± 9.9 month follow-up, 22 cases (34.4%) suffered from recurrence. All 64 patients were classified into two groups, namely, group 1 (with recurrence) and group 2 (without recurrence). A statistically significant difference was reported between the two groups in empirical superior vena cava (SVC) isolation (28.6 vs. 60.5%, *p* = 0.019). LVAs in LA (63.6 vs. 45.2%, *p* = 0.162), linear ablation (54.5 vs. 45.2%), and non-PV triggers (9.1 vs. 16.7%, *p* = 0.707) had no significant difference between the two groups. The recurrence rate among the groups defined by procedure characteristics is demonstrated in Table 3 and Figure 3. Procedural characteristics of empirical and non-empirical SVC isolation groups are demonstrated in Table 4.

**TABLE 3:** Procedural characteristics of the recurrent and non-recurrent groups.

	Recurrent group (*n* = 22)	Non-recurrent group (*n* = 42)	*p*-value
Low voltage in LA *n* (%)	14 (63.6)	19 (45.2)	0.162
Anterior wall *n* (%)	12 (54.5)	17 (40.5)	0.283
Posterior wall *n* (%)	1 (4.5)	8 (19.0)	0.196
Roof *n* (%)	9 (40.9)	4 (9.5)	0.007
Septum *n* (%)	3 (13.6)	1 (2.4)	0.113
Substrate modification *n* (%)	10 (45.4)	15 (35.7)	0.448
Linear ablation *n* (%)	12 (54.5)	19 (45.2)	0.479
Anterior wall *n* (%)	11 (50.0)	12 (28.6)	0.090
Posterior wall *n* (%)	1 (4.5)	7 (16.7)	0.245
Roof *n* (%)	7 (31.8)	7 (16.7)	0.208
CTI *n* (%)	9 (40.9)	13 (31.0)	0.426
AT *n* (%)	12 (50.4)	16 (38.1)	0.208
Roof-dependent reentry *n* (%)	2 (9.1)	4 (9.5)	>0.999
MA-dependent reentry *n* (%)	6 (27.3)	6 (14.3)	0.312
TA-dependent reentry *n* (%)	2 (9.1)	2 (4.8)	0.603
Focal *n* (%)	1 (4.5)	3 (7.1)	>0.999
Non-PV triggers *n* (%)	2 (9.1)	7 (16.7)	0.707
Empirical SVC isolation (%)	6/21 (28.6)	23/38 (60.5)	0.019

AT, atrial tachycardia; CTI, cavo-tricuspid isthmus; LA, left atrium; MA, mitral annulus; SVC, superior vena cava; TA, the tricuspid annulus.

**TABLE 4:** Procedural characteristics of empirical and non-empirical superior vena cava (SVC) isolation groups.

	Empirical SVC isolation (*n* = 29)	Non-empirical SVC isolation (*n* = 30)	*p*-value
Low voltage in LA *n* (%)	13 (44.8)	19 (63.3)	0.154
Anterior wall *n* (%)	12 (41.4)	16 (53.3)	0.358
Posterior wall *n* (%)	2 (6.9)	6 (20.0)	0.142
Roof *n* (%)	5 (17.2)	8 (26.7)	0.383
Septum *n* (%)	3 (10.3)	1 (3.3)	0.284
Substrate modification *n* (%)	9 (31.0)	16 (53.3)	0.083
Linear ablation *n* (%)	10 (54.5)	20 (66.7)	0.013
Anterior wall *n* (%)	6 (20.7)	16 (53.3)	0.010
Posterior wall *n* (%)	3 (10.3)	4 (13.3)	0.723
Roof *n* (%)	6 (20.7)	8 (26.7)	0.590
CTI *n* (%)	8 (27.6)	12 (40.0)	0.314
AT *n* (%)	6 (20.7)	21 (70.0)	<0.001
Roof-dependent reentry *n* (%)	0 (0.0)	6 (20.0)	0.011
MA-dependent reentry *n* (%)	1 (3.4)	11 (36.7)	0.002
TA-dependent reentry *n* (%)	2 (6.9)	1 (3.3)	0.533
Focal *n* (%)	2 (6.9)	2 (6.7)	0.972
Non-PV triggers *n* (%)	4 (13.8)	1 (3.3)	0.149
FO trigger (%)	2 (6.9)	1 (3.3)	0.533
CT trigger (%)	2 (6.9)	0 (0.0)	0.305

AT, atrial tachycardia; CT, crista terminalis; CTI, cavo-tricuspid isthmus; FO, fossae ovalis; LA, left atrium; MA, mitral annulus; SVC, superior vena cava; TA, the tricuspid annulus.

#### Predictors for recurrence

**FIGURE 4:**
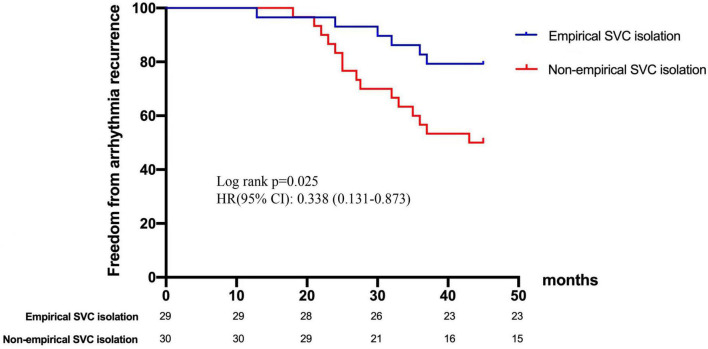
Freedom from arrhythmia recurrence after repeat ablation procedure. Kaplan-Meier curve demonstrates significant difference in recurrence between empirical and non-empirical SVC isolation groups. SVC, superior vena cava.

In terms of age, gender, hypertension, coronary heart disease, CHA_2_DS_2_-VASc score, and left atrial dimeter, no significant difference was found between the recurrent and non-recurrent groups. Univariate analysis indicated that the two groups significantly differed in the type of AF, diabetes, and empirical SVC isolation. Multivariate regression analysis demonstrated that empirical SVC isolation was an independent predictor of sinus rhythm maintenance (*p* = 0.009, 95% CI: 1.64–32.8). Univariate and multivariate analyses for factors predicting recurrence after the re-ablation procedure are displayed in Table 5. Time-to-event analyses are shown in Figure 4. The Kaplan-Meier survival curve showed significant difference in maintenance of SR between empirical and non-empirical SVC isolation groups (79.3 vs 50.0%; HR: 0.338; 95% CI: 0.131–0.873; *p* = 0.025).

**TABLE 5:** P Univariate and multivariate analyses for factors predicting recurrence post the re-ablation procedure.

	Univariate		Multivariate	
	95% CI	*P*	95% CI	*P*
Non-paroxysmal AF	1.07–9.58	0.038	0.867–21.26	0.074
Diabetes	0.024–0.731	0.02	0.06–5.91	0.663
CHA_2_DS_2_-VASc	0.998–2.144	0.051	0.63–1.80	0.811
LVDd	0.992–1.285	0.065	0.957–1.338	0.147
SVC isolation	1.88–19.9	0.003	1.64–32.8	0.009

LVDd, left ventricular diastolic dimeter; SVC, superior vena cava.

#### Complications

No severe complications were detected until discharge and during follow-up.

## Discussion

This retrospective study demonstrated that 22% of the repeat AF patients were PVI non-responders. Paroxysmal AF, without diabetes mellitus, and empirical SVC isolation were associated with freedom from recurrence of atrial tachycardias, and only empirical SVC isolation was an independent predictor of the maintenance of SR.

### Pulmonary vein reconnection and atrial fibrillation recurrence

Haissaguerre found that the occurrence of AF was closely associated with triggering foci, which are typically located in PVs. Therefore, PVI has become the cornerstone of ablation for AF. Prior studies suggest that the recurrence of AF is associated with the reconnection of PVs. The primary reasons were as follows: (1) the likelihood of finding four isolated PVs during the second ablation was relatively rare; and (2) re-isolation of the gaps improved the outcomes of ablation (11). Prior studies demonstrated a low incidence of complete PV isolation during the second ablation (12). However, with the wide application of a contact-force sensing catheter (CFSC) and second-generation cryoballoon, the efficiency of PVI significantly increased (13–16). Currently, the incidence of finding four isolated PVs during the second AF ablation is much higher than before. De Pooter et al. (17) reported that in a CSFC-guided PVI investigation (CLOSE study), 62% of recurrent patients were PVI non-responders, suggesting that the reconnection of PVs was no longer a major factor for the recurrence after initial ablation. This present study found that 78% of patients with recurrent AF had one or more PVs reconnected during the second procedure, which was higher than recent data. The possible reason may be due to a high proportion (73.2%) of initial procedures guided by non-contact-force sensing catheters. With the development of technology and the prevalence of CFSC, the proportion of PVI non-responders will gradually increase. Therefore, optimal strategies for the second ablation procedure for these types of patients merit further research.

### Fibrosis, linear ablation of left atrium, and atrial fibrillation recurrence

Fibrosis of the atrium was closely associated with the occurrence and maintenance of AF and was considered an important substrate for the maintenance of non-paroxysmal AF (18, 19). Prior studies suggested that patients with severe left atrial fibrosis had a lower chance of maintaining SR post-ablation (20–22). It was reported that low voltage distribution is highly consistent with the fibrotic area detected by cardiac magnetic resonance (23). Voltage mapping during SR is typically applied to reflect the fibrosis of LA. Therefore, a high-density voltage mapping-guided substrate modification was generally used in this study. The linear ablation of the left atrium was performed according to the distribution of low voltage areas. For patients without a low voltage in the left atrium, no additional linear ablation was performed to avoid any iatrogenic atrial tachycardia. The strategy for individual substrate modification was described in our previous study (9). The present study demonstrated that other strategies, including linear ablation and substrate modification did not improve the outcomes of the repeated procedure. We speculate the possible reasons as follows: although substrate modification could potentially improve the outcome of patients with “diseased” left atrium, such benefit might be diluted in the whole AF population, especially when the sample size was not powerful enough to support the benefit.

### Non-PV triggers and recurrence of atrial fibrillation

**FIGURE 5:**
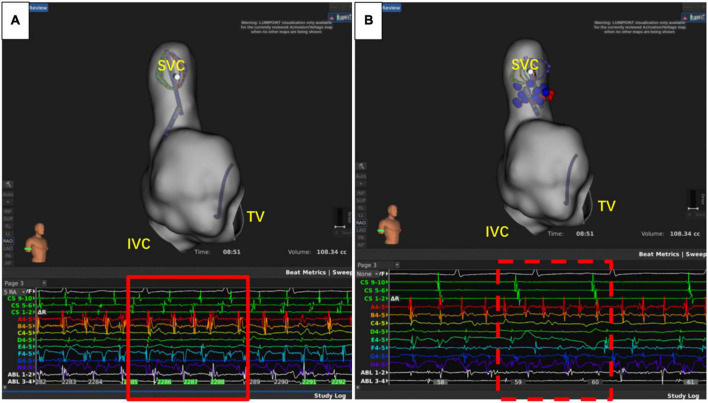
Superior vena cava (SVC) triggers atrial fibrillation. **(A)** Atrial fibrillation before isolation of SVC. **(B)** After SVC isolation, sinus rhythm was restored in the atrium, while the atrial fibrillation rhythm was sustained in SVC. IVC, inferior vena cava; TV, tricuspid valve.

The recurrence of AF is also closely related to dormant non-PV triggers (24), especially in patients with durable PVI. The SVC, fossae ovalis, crista terminalis, coronary vein, and left atrial appendage are common non-PV triggers (25). About 10–20% of AF patients had non-PV triggers, and this was associated with the recurrence of AF (26, 27). Therefore, it is of great significance to eliminate non-PV triggers (28). Currently, the commonly used strategies to reveal non-PV triggers include intravenous infusion of isoproterenol, adenosine triphosphate, and burst stimulation in the atrium (26, 28). However, the detection ratio of non-PV triggers was relatively low. In this study, only nine cases (14.1%) had definite non-PV triggers, including five cases of SVC (Figure 5), one case of crista terminalis, and three cases of fossae ovalis, suggesting that SVC was the most common non-PV trigger in the Chinese population. This is consistent with the results reported by prior studies (29, 30). Thus, patients with concealed non-PV triggers in the Asian population were most likely to be with SVC triggers and could benefit from empirical SVC isolation, which is in consistency with the results from other population. Additionally, unlike other non-PV triggers elimination, SVC isolation is a more commonly applied technique with exact endpoint, making the ablation results more replicable and reliable even when the non-PV triggers in uninducible., and superior vena cava isolation is proved to be safe and feasible (31, 32). Our study demonstrated that empirical SVC isolation was the only independent predictor of recurrence after the second ablation of AF, suggesting that empirical SVC isolation improved the outcome of re-ablation in patients with all PVs isolated.

## Limitations

Our study has some limitations. First, this was a retrospective study with small sample size, which led to potential selection bias. Second, no data on serum markers of atrial fibrosis were available. Third, a stronger follow-up strategy employing a 7-day Holter ECG or implanted loop recorder may be needed to detect the recurrence of asymptomatic atrial arrhythmia. Fourth, other empirical interventions of dormant non-PV triggers were not adopted and compared in this study. Further large-scale prospective studies with longer follow-up times are needed to validate the efficacy and safety of empirical SVC isolation in PVI non-responders.

## Conclusion

During the repeat procedure of AF ablation, 22% were identified as PVI non-responders. Empirical SVC isolation may improve the outcome of re-ablation.

## Data availability statement

The original contributions presented in this study are included in the article/Supplementary material, further inquiries can be directed to the corresponding author.

## Ethics statement

The studies involving human participants were reviewed and approved by Ethics Committee of The First Affiliated Hospital of Nanjing Medical University. The patients/participants provided their written informed consent to participate in this study.

## Author contributions

All authors listed have made a substantial, direct, and intellectual contribution to the work, and approved it for publication.
